# Neuroimaging and Transcriptomic Insights Into Iron Accumulation and Glymphatic Dysfunction in Olfactory Dysfunction

**DOI:** 10.1002/cns.70677

**Published:** 2026-01-16

**Authors:** Chantat Leong, Jixin Luan, Ruisi Wang, Manxi Xu, Hongwei Yu, Li Zhu, Ni Shu, Gaoxiang Ouyang, Hui Xia, Guolin Ma, Zhen Yuan

**Affiliations:** ^1^ Centre for Cognitive and Brain Sciences University of Macau Macau SAR China; ^2^ Faculty of Health Sciences University of Macau Macau SAR China; ^3^ Department of Radiology China‐Japan Friendship Hospital Beijing China; ^4^ China‐Japan Friendship Hospital (Institute of Clinical Medical Sciences) Chinese Academy of Medical Sciences & Peking Union Medical College Beijing China; ^5^ Department of Radiology General Hospital of Ningxia Medical University Yinchuan China; ^6^ State Key Laboratory of Cognitive Neuroscience and Learning Beijing Normal University Beijing China; ^7^ Department of Engineering Electromagnetic Field and its Application Institute of Electrical Engineering Chinese Academy of Sciences Beijing China; ^8^ Zhuhai UM Science & Technology Research Institute University of Macau Zhuhai China

**Keywords:** cerebrospinal fluid flow, functional MRI, gene expression, glymphatic function, iron accumulation, olfactory dysfunction, quantitative susceptibility mapping

## Abstract

**Background:**

Olfactory dysfunction (OD) is clinically linked to inflammation and neurotoxin accumulation, yet the underlying neurobiological mechanisms remain largely unclear. Understanding how glymphatic function, iron dysregulation, and transcriptomic signatures contribute to OD may reveal new biomarkers and mechanisms of recovery.

**Methods:**

A multimodal MRI framework integrating BOLD–CSF coupling, quantitative susceptibility mapping (QSM), and transcriptomic profiling was applied to post‐viral (PVOD), post‐traumatic (PTOD), and healthy control (HC) groups. Iron accumulation was quantified with QSM and linked to gene expression using partial least squares regression, followed by GO and protein–protein interaction analyses.

**Results:**

PVOD showed significantly increased iron accumulation in the right inferior frontal and temporal cortices, regions related to olfactory memory and recognition. Transcriptomic associations indicated that iron deposition correlated with genes involved in neuronal organization, axon development, synapse formation, and intracellular signaling. PVOD also demonstrated enhanced glymphatic activity, reflected by stronger BOLD–CSF coupling compared to HC and PTOD. Patients with complete recovery exhibited the strongest coupling, suggesting improved neurotoxin clearance.

**Conclusion:**

OD is characterized by abnormal iron accumulation and altered glymphatic function, accompanied by transcriptional signatures supporting neuroplasticity. Enhanced glymphatic clearance and neuronal remodeling may facilitate recovery after viral injury, offering potential biomarkers for OD diagnosis and prognosis.

## Graphical abstract

Exploring iron accumulation and lymphatic function can assess the expression and change of neurotoxins in olfactory dysfunction (OD). This study further combines transcriptome analysis to demonstrate the biological pathways linked to neurotoxins, offering new insights into OD's neurobiology.

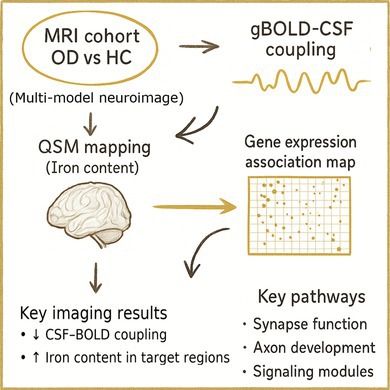

## 1 Introduction

Olfactory dysfunction (OD) is characterized by anosmia (function loss), hyposmia (sensitivity decrease), dysosmia (alterations in odor) and phantosmia (hallucination) [1]. In particular, post‐viral OD (PVOD) has received widespread attention especially after COVID‐19. Besides traumatic brain injury and sinonasal inflammatory processes, PVOD is one of the leading causes of anosmia [2]. Meanwhile, it was discovered that the recovery of OD depended on the plasticity of brain functions of the olfactory bulb and central olfactory pathways [3, 4]. To date, neuroimaging studies have been performed to inspect the brain's structural and functional abnormalities in OD. Magnetic resonance imaging (MRI) findings showed that brain morphological changes were mainly detected in the olfactory nerve, olfactory bulb, and olfactory cortices including the hippocampus, thalamus, and cerebellum [5, 6]. Functional MRI (fMRI) studies illustrated that reduced brain activity in OD subjects occurred primarily in the primary olfactory region (POC), orbitofrontal cortex (OFC), and insular cortex [7, 8, 9]. Besides, the structural and functional connectivity as well as the microstructural properties of white matter and brain activity also exhibited the difference between OD and healthy control (HC) groups [10, 11, 12, 13]. In OD, these structural and functional neural activity or connectivity impairments are thought to be associated with the accumulation of neurotoxins [14]. Yet the mechanisms and performance of neurotoxins in OD, whether neurotoxins are the cause of neuronal damage or a potential biomarker, remain unknown.

The glymphatic system is recognized as regulating the balance of inflammatory factors in the central nervous system [15]. Accumulating evidence has demonstrated that an impaired glymphatic system corresponds to decreased removal of toxins within the extracellular interstitial space, causing an increased accumulation of protein and waste products. More importantly, fMRI has captured the coupling between global bold signals and CSF influx that is the indicator of the rhythm of glymphatic function [16]. In particular, reduced coupling rhythms were detected in neurodegenerative diseases such as Alzheimer's disease or Parkinson's disease [17, 18, 19]. Therefore, it is rational to assume that the coupling rhythm might be altered in OD due to the interplay between inflammation and the glymphatic system. For example, mild COVID‐19 patients revealed decreased asymmetric bilateral glymphatic function in the brain after 4 months of recovery [20].

Besides, different from the glymphatic system, recent animal studies illustrated that progressive iron overload might disrupt olfactory function, trigger lipid oxidation and promote neuronal apoptosis [21]. Iron homeostasis is essential to maintain the balance of iron concentration in the brain, preventing the neurotoxic effects of excess free iron. Once iron metabolism is altered, pathological proteins can bind to iron and react with hydrogen peroxide to generate hydroxyl radicals, ultimately triggering neuroinflammatory responses and inducing ferroptosis [22]. The ferroptosis pathway can lead to mitochondrial dysfunction and DNA damage [23]. Thus, abnormal iron metabolism can serve as a biomarker of neuronal death and loss, in which iron binds to transferrin in epithelial cells and is internalized via the transferrin–transferrin receptor pathway. It then enters the brain through the blood–brain barrier (BBB) [24]. Quantitative susceptibility mapping (QSM) is a MRI technique that measures the spatial distribution of magnetic susceptibility of tissues, providing the ability to calculate the different magnetic susceptibility of various tissues [25]. In particular, QSM is sensitive to iron. Herein, the iron deposition index captured by QSM might have the ability to detect neuronal damage in OD.

Recently, radiomics analysis, which combines neuroimaging information with the transcriptome using whole‐brain gene expression profiles, has shown advantages in studying the neural mechanisms of various brain disorders [26, 27, 28]. Regional gene performances have been carefully explored by co‐localizing regional genes with neuroimaging biomarkers [29, 30]. It is hypothesized that alterations in the neurological dimension of OD are detected by glymphatic function and iron accumulation, which further investigates the spatial associations between disease neuroimaging patterns and gene expression in the brain. This may help to understand the genetic mechanisms behind the biomarkers of OD images and thus elucidate the characterization of OD more comprehensively.

To test the hypothesis, OD patients were first divided into PVOD (mainly COVID‐infected patients) and PTOD groups to identify the endogenous and exogenous olfactory differences between the two groups as compared to those of HC. Subsequently, we proposed a multimodal and multiscale MRI approach to examine the changes in glymphatic clearance capacity and microscale molecular functional manifestations. In particular, BOLD‐CSF coupling was generated to access the glymphatic function in PVOD while QSM was conducted to quantify the iron levels in the brain. Besides, the correlation between the iron accumulation and gene expression in OD was assessed by applying partial least squares (PLS) regression via aligning the atlas in QSM data with Allen Human Brain Atlas (AHBA) transcriptomic data. Finally, gene ontology (GO) enrichment analysis and protein–protein interaction (PPI) analysis were carried out to determine the biological pathways and protein networks that were linked to the aberrant iron accumulation in PVOD.

## 2 Methods

### 2.1 Subjects

Participants for the present study included 60 HC, 69 PVOD, and 20 PTOD patients, with mean ages of 50.5 ± 18.6, 37.1 ± 10.8, and 38.5 ± 10.8 years, and male/female ratios of 17/43, 31/38, and 7/13, respectively. PVOD and PTOD patients were continuously recruited between February 2023 and December 2023 from the Center for Smell and Taste Disorders with the Department of Otorhinolaryngology, Head and Neck Surgery at China‐Japan Friendship Hospital. This work followed the Declaration of Helsinki and the protocol was approved by the Ethics Committee of both the University of Macau and China‐Japan Friendship Hospital (No. 2022‐KY‐181). Informed consent was acquired from all participants.

The inclusion criteria for the PVOD were: (1) any one of the following symptoms such as anosmia, hyposmia, parosmia, and phantosmia, (2) confirmation of viral infection at symptom onset, (3) persistence of OD for at least 1 month without intervention, and (4) patient‐reported symptoms or TDI score confirming decreased olfaction. By contrast, the criteria for PTOD consisted of: (1) any one of the following symptoms including anosmia, hyposmia, parosmia, and phantosmia, (2) histories of head trauma, (3) persistence of OD for at least 1 month without intervention, and (4) patient‐reported symptoms or TDI score confirming decreased olfaction. The exclusion criteria for both the PVOD and PTOD were: (1) histories of pregnancy or lactation, and (2) other potential causes of OD, such as nasal obstruction, sinus infections, or smoking.

Participants from HC group were recruited from the local communities between February 2018 and October 2019, prior to the onset of the study period. Participants needed to go through the same MRI protocol as the patient cohort without conducting olfactory or neuropsychological evaluations. None of the HC participants reported a history of olfactory or cognitive deficits, or neurological impairments at the time of the MRI.

All participants underwent the routine MRI examinations. However, the inclusion of their QSM sequences and CSF signals in this study was determined by the completion of 3D gradient‐echo sequences and the quality assessment of single‐shot gradient‐recalled echo‐planar imaging. The analyses included QSM/CSF measurements for HC (*n* = 60/50), PVOD (*n* = 54/56), and PTOD (*n* = 19/20) groups. The demographic data for all OD patients were collected by using the Sniffin' Sticks test. The Sniffin' Sticks test was able to assess the three core olfactory components: odor threshold (T), odor discrimination (D), and odor identification (I). For patients with PVOD, we conducted both telephone follow‐ups and on‐site visits. During telephone follow‐ups, participants were asked to self‐report their recovery status by selecting among three options: Partial recovery, complete recovery, or no recovery.

### 2.2 Image Acquisition

The hospital used a Discovery MR750 3.0‐T scanner (General Electric, Milwaukee, WI, USA) with an eight‐channel head coil to perform MR scanning, including a T1, rs‐fMRI, and QSM scans. The MRI parameters used were: (1) T1: repetition time (TR) = 6.7 ms, echo time (TE) = 2.9 ms, flip angle = 12°, slice thickness = 1 mm, field of view (FOV) = 256 × 256 mm, and voxel size = 1 × 1 × 1 mm^3^; (2) rs‐fMRI: TR = 2000 ms, TE = 30 ms, flip angle = 90°, slice thickness = 3.5 mm, slice spacing = 0.7 mm, FOV = 224 mm × 224 mm, voxel size = 3.5 × 3.5 × 3.5 mm^3^, and 240 measurements; (3) QSM: TE1st/ΔTE/TE8th = 3.19 ms/2.37 ms/19.77 ms, TR = 22.9 ms, bandwidth = 62.5 Hz/pixel, slice thickness = 1.0 mm, FOV = 256 mm × 256 mm, and voxel size = 1 × 1 × 1 mm^3^.

### 2.3 Data Processing

**FIGURE 1:**
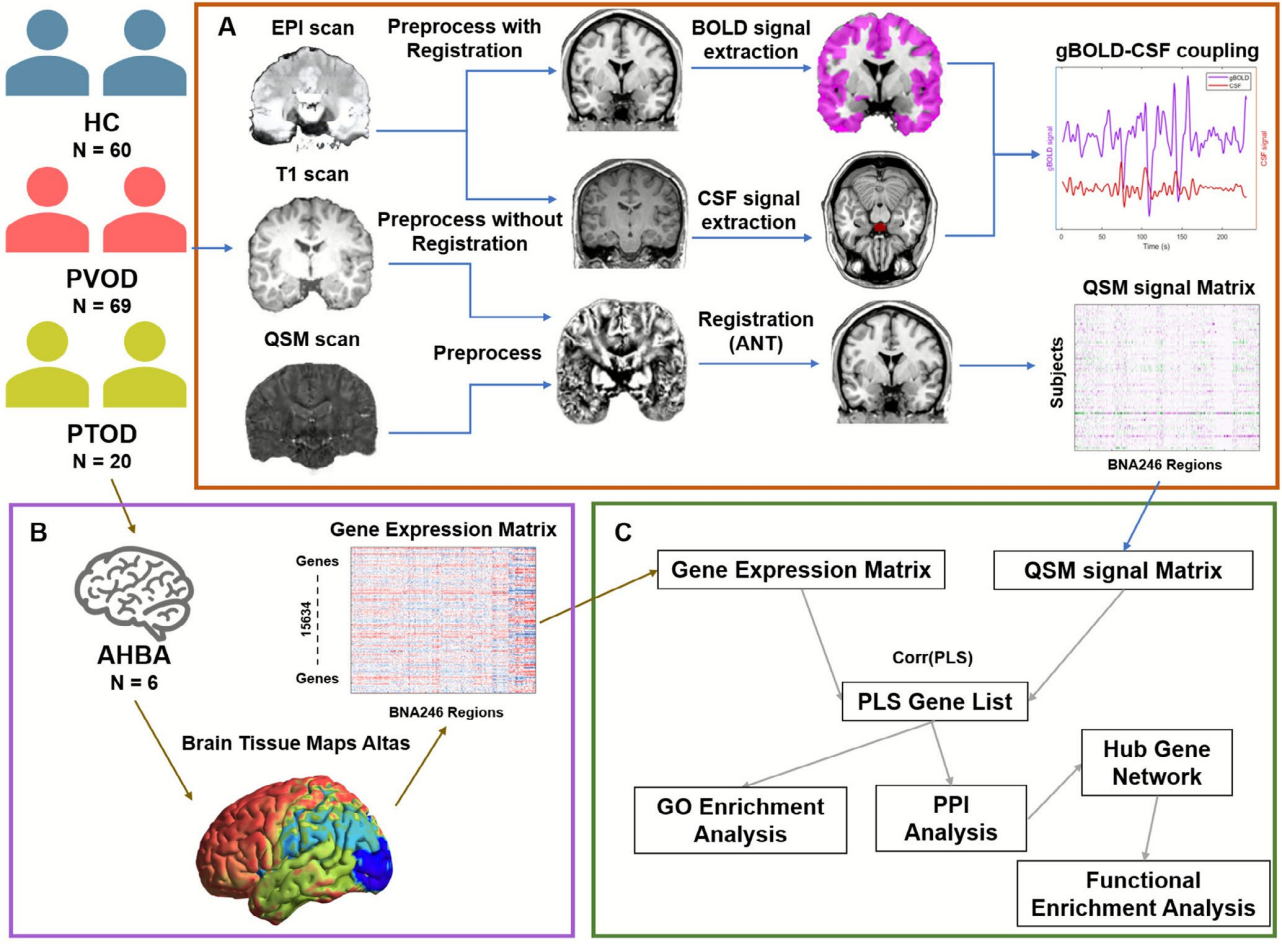
Workflow of multi‐omics data collection and preprocessing. (A) The cohort of 149 subjects was divided into HC (*N* = 60), PVOD (*N* = 69), PTOD (*N* = 20), T1 weighted MRI, rs‐fMRI, and QSM scans. The global BOLD signal was extracted from cortical regions (purple color). The CSF signal region was extracted from the bottom slice of the CSF segmented rs‐fMRI image (red color). Cross‐correlation analysis was applied to the BOLD and CSF signal fluctuations time series. 3D multi‐echo GRE images were preprocessed and calculated to generate the QSM map. Then, it was registered to the BNA246 atlas and segmented. (B) The gene expression data were from 6 postmortem donors from AHBA (http://human.brain‐map.org). Whole brain gene expression has been registered to MNI space. BNA246 atlas. Gene expression was then extracted to produce a 246 (regions) × 15,634 (genes) gene expression matrix according to BNA 246 atlas. (C) Partial least squares (PLS) regression was used to identify imaging transcriptomic associations between QSM signals and gene expression. GO enrichment and PPI analysis further revealed the involvement of biological processes and hub gene networks.

The data processing pipeline was presented in Figure 1.

### 2.4 The Rs‐fMRI Preprocess

All the preprocessing steps of rs‐fMRI were preprocessed in the Data Processing Assistant for Resting‐State fMRI (DPABI) [31]. To extract CSF signal, the preprocessing pipeline was as follows. First, the first five measurements were discarded to avoid distortion effects. Second, slice timing and motion correction were adjusted for temporal and spatial inconsistency. Third, data drift was detrended using quadratic time trend removal and a 0.01–0.1 Hz bandpass filter. Fourth, spatial smoothing was conducted with a 4 mm full‐width half‐maximum (FWHM) kernel. Afterward, rs‐fMRI and T1 image were co‐registered through Statistical Parametric Mapping 12 tool (SPM12) [32], and T1 image was further segmented into a CSF mask. The CSF signals were extracted from the nonzero bottom slice of the CSF mask [16]. The time series of CSF signals were further averaged and normalized across time series for each subject.

To extract BOLD signal, slice timing, motion correction, temporal filter, and trend removal processes were not changed. Subsequently, the co‐registered process used the Diffeomorphic Anatomical Registration Through Exponentiated Lie Algebra (DARTEL) function in DPABI to convert rs‐fMRI from the individual's space to the Montreal Neurological Institute (MNI‐152) space. Finally, rs‐fMRI data were further normalized and smoothed with 4 mm FWHM. The BOLD data were extracted from gray matter (GM) cortical regions [33] by using the automated Brainnetome Atlas (BNA) 246 [34], and averaged across all GM voxels. The time series of BOLD signals were normalized across the whole time series for each subject. Of these, 8 PVOD and 9 HC were excluded due to head movements.

### 2.5 Quantify the Coupling Between BOLD and CSF Signal

The BOLD‐CSF coupling was quantified by cross‐correlation functions, which compute the Pearson correlations between two signals from −12 s to 12 s time lags for each subject. Time lag was chosen based on the peaks of negative coefficients that occurred most frequently across all study subjects. The correlation coefficients were selected according to the time lag. To assess the statistical significance of the coupling, a permutation test was conducted on the correlation coefficients with 3000 permutations. The *p*‐value was calculated as the percentage of the true data that was lower than the randomized data [16, 17]. Furthermore, cross‐correlation of the negative derivatives of BOLD and CSF signals was also performed to confirm that the negative peak coefficients between the BOLD and CSF signals aligned [16, 17]. The confounder effects of BOLD‐CSF coupling were investigated, including gender, age, duration and so on. When these effects were significant, they were included as covariates in subsequent analyses.

### 2.6 QSM Preprocess and Regional QSM Extraction

QSM images were derived from phase and magnitude data acquired using a three‐dimensional multi‐echo gradient echo sequence. All data processing was performed in MATLAB 2018 using the STI Suite toolbox (v3.0; https://people.eecs.berkeley.edu/~chunlei.liu/software.html). Phase unwrapping and background phase removal were simultaneously performed using HARPERELLA (HArmonic PhasE REmovaL using LAplacian operator) [35]. Brain masks were generated from the echo magnitude images. Background field effects were removed using V‐SHARP (Variable‐kernels Sophisticated Harmonic Artifact Reduction for Phase data) [36] to obtain local field maps containing tissue susceptibility information. The STAR (Streaking Artifact Reduction) algorithm was applied [37]. The algorithm through a two‐level regularization approach generates the QSM images. QSM image normalization was achieved using ANTs (Advanced Normalization Tools; https://stnava.github.io/ANTs/) software. The first‐echo magnitude images were co‐registered to T1 images, after which the QSM images were normalized to MNI space using the affine transformation matrix obtained from this registration process. The ROI signals were extracted from QSM images using the BNA246 atlas via ANTs. The confounder effects of QSM signals were also tested and adjusted as covariates if significant.

### 2.7 Regional Microarray Expression Data Extraction

Regional microarray expression data were obtained from the AHBA, which consisted of six postmortem brains [38]. The Abagen toolbox was used to process and map the transcriptomic data to BNA 246 atlas. Gene probes in the AHBA dataset were re‐annotated and probes with unreliable gene matches were eliminated [39]. The re‐annotated probes were further filtered based on their intensity relative to the background noise level of less than 50%.

Tissue samples were then assigned to brain regions based on the nearest region within a radius of 2 mm using the corrected MNI coordinates. If a brain region was not assigned to any sample, the closest sample to the region's centroid was used to represent that region. Gene expression values were then normalized for each donor across regions using the sigmoid function, and further averaged across donors. Since the AHBA contained only left hemisphere data for two subjects, only the left hemisphere region was selected for analysis in the correlational analysis.

### 2.8 Quantify the Transcriptomic Correlations of OD's QSM

PLS regression was applied to quantify the correlation coefficients by using transcriptome measurements to predict regional QSM data in PVOD [40]. The PLS components were calculated as the linear combination of the weighted gene expression scores that covary the most with QSM changes. As the number of components increases, subsequent components explain progressively less variance in the dependent variable (QSM changes). In our analysis, 15 components were quantified with respect to the relative variance explained by each component. After 1000 permutation tests, PLS components were selected based on a permutation *p*‐value threshold of *p*_boot_ < 0.05. The PLS component for further analysis was chosen according to the highest explained variance among all components; the component with the highest explained variance was selected. The bootstrapping method was used (randomly substituting and resampling 123 brain regions) to estimate the error of each gene, and a *Z*‐score was calculated using the ratio of the weight of each gene to the standard error [41]. The list of genes was screened for enrichment analysis within ±2.8 (*p*_fdr_ < 0.05).

### 2.9 GO Enrichment Analysis and PPI Analysis

To explore whether these genes map to common related biological pathways, we performed the enrichment analysis of significant weighted genes using the clusterProfiler package in R. GeneID was converted to entrez genes and reduced the unrecognized geneID. Then, GO enrichment analysis was performed with the Benjamini–Hochberg FDR correction (*q* < 0.05). The significant genes were further selected to construct PPI networks by using STRING (version 11.5) (https://www.string‐db.org/). The interactions were shown with an interaction score higher than 0.7 among the list of target genes by using Cytoscape [42]. The CytoHubba plugin (http://apps.cytoscape.org/apps/cytohubba) identified the hub gene network. The hub gene network underwent functional enrichment analysis.

### 2.10 Statistical Analysis

A *t*‐test and Pearson correlation were utilized to account for the effect of confounding factors. A one‐way ANOVA was then performed to compare the intergroup differences between BOLD‐CSF coupled and QSM signals, and FDR corrections were applied. Additionally, Pearson correlation was employed to investigate the relationship between total Sniffin' Sticks scores (SS total) and the BOLD‐CSF coupling or QSM signals.

In addition, PVOD was categorized as fully recovered, partially recovered, and unrecovered after the follow‐up investigation. The Kruskal–Wallis Test further compared the BOLD‐CSF coupling to the QSM signals and correlated them to the total SS in all three conditions using Spearman's Rank Correlation. Summary‐level processed data supporting these analyses are provided in the Data S1.

## 3 Results

### 3.1 Demographic and Clinical Characteristics

The demographic and clinical characteristics were summarized in Table 1.

**TABLE 1:** Demographic and clinical characteristics of PVOD, PTOD, and HC groups.

Characteristics	HC (*n* = 60)	PVOD (*n* = 69)	PTOD (*n* = 20)	*F*‐value or *T*‐value	*p*	
Anosmia		*n* = 23	*n* = 11			
Hyposmia		*n* = 39	*n* = 9			
Parosmia		*n* = 7	*n* = 0			
Age	50.5 ± 18.6	37.1 ± 10.8	38.5 ± 10.8	14.66	< 0.001**	
Sex (M/F)	43/17	31/38	7/13	1.93	0.149	
Duration (day)		267.8 ± 475.3 (*n* = 54)	245.6 ± 408.6 (*n* = 20)	−0.33	0.74	
SS_T	\	2.9 ± 2.57 (*n* = 61)	1.58 ± 2.23		0.045*	PVOD > PTOD
SS_D	\	7.23 ± 4.77 (*n* = 61)	3.45 ± 3.96		0.002*	PVOD > PTOD
SS_I	\	8.85 ± 4.99 (*n* = 61)	4.2 ± 4.07		< 0.001**	PVOD > PTOD
SS_total	\	18.98 ± 11.2 (*n* = 61)	9.23 ± 8.58		< 0.001**	PVOD > PTOD

Abbreviations: HC, healthy control; M/F, male/female; *n*, number of subjects; PTOD, post‐traumatic olfactory dysfunction; PVOD, post‐viral olfactory dysfunction; SS_D, Sniffin' sticks discrimination test; SS_I, Sniffin' sticks identification test; SS_T, Sniffin' sticks threshold test; SS_total, Sniffin' sticks total score. Significance of * and ** indicates *p* < 0.05 and *p* < 0.001, respectively.

### 3.2 The BOLD‐CSF Coupling Alteration

**FIGURE 2:**
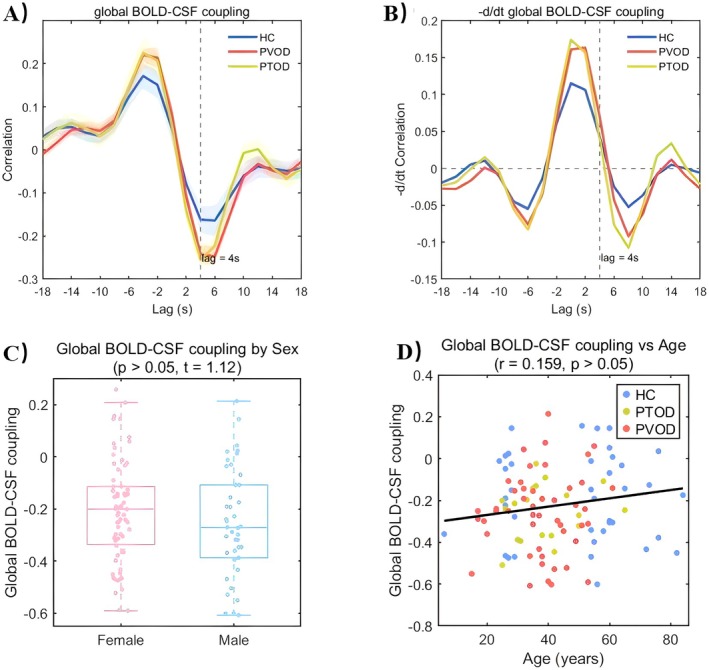
Three groups of BOLD‐CSF coupling strengths and their associations with age and gender. (A) Cross‐correlations between BOLD and CSF signals for PTOD, PVOD, and HC. (B) Negative derivatives of BOLD and CSF signals. Vertical dashed lines indicate the negative peak at a +4 s time lag. (C) Comparison of BOLD‐CSF coupling between males and females, showing no significant sex difference. (D) Correlation between BOLD‐CSF coupling and age, showing no significant association. Error bars represent standard deviation (SD). Blue, red, and yellow indicate HC, PVOD, and PTOD groups, respectively.

Among the 3 groups, the negative peak of coupling appeared on the interactions with a time lag of +4 s (*r* = −0.256 ± 0.128 for PVOD; *r* = −0.248 ± 0.191 for PTOD; *r* = −0.165 ± 0.223 for HC; *ps* < 0.05 with permutation test, Figure 2A). The negative derivative of coupling was also 0 at the +4 s time lag, which is indicative of glymphatic function (Figure 2B). As no significant differences in coupling strength were found between sexes (*t* = 1.14, *p* = 0.257, Figure 2C) and no correlation was observed with age (*r* = 0.71, *p* = 0.077, Figure 2D). The BOLD‐CSF coupling strength was analyzed using ANOVA among the three groups (PVOD, PTOD, and HC), where significant differences were revealed (*F* = 3.13, *p* = 0.048). Post hoc Tukey's test showed significantly higher coupling strength in the HC compared to the PVOD group (*p* = 0.049), with no significant differences between other group comparisons (*ps* > 0.05). Additional confounding comparisons (including head motion, disease duration, corticosteroid treatment, and olfactory training) are detailed in Figures S1 and S2.

### 3.3 Alterations of QSM Signal

**FIGURE 3:**
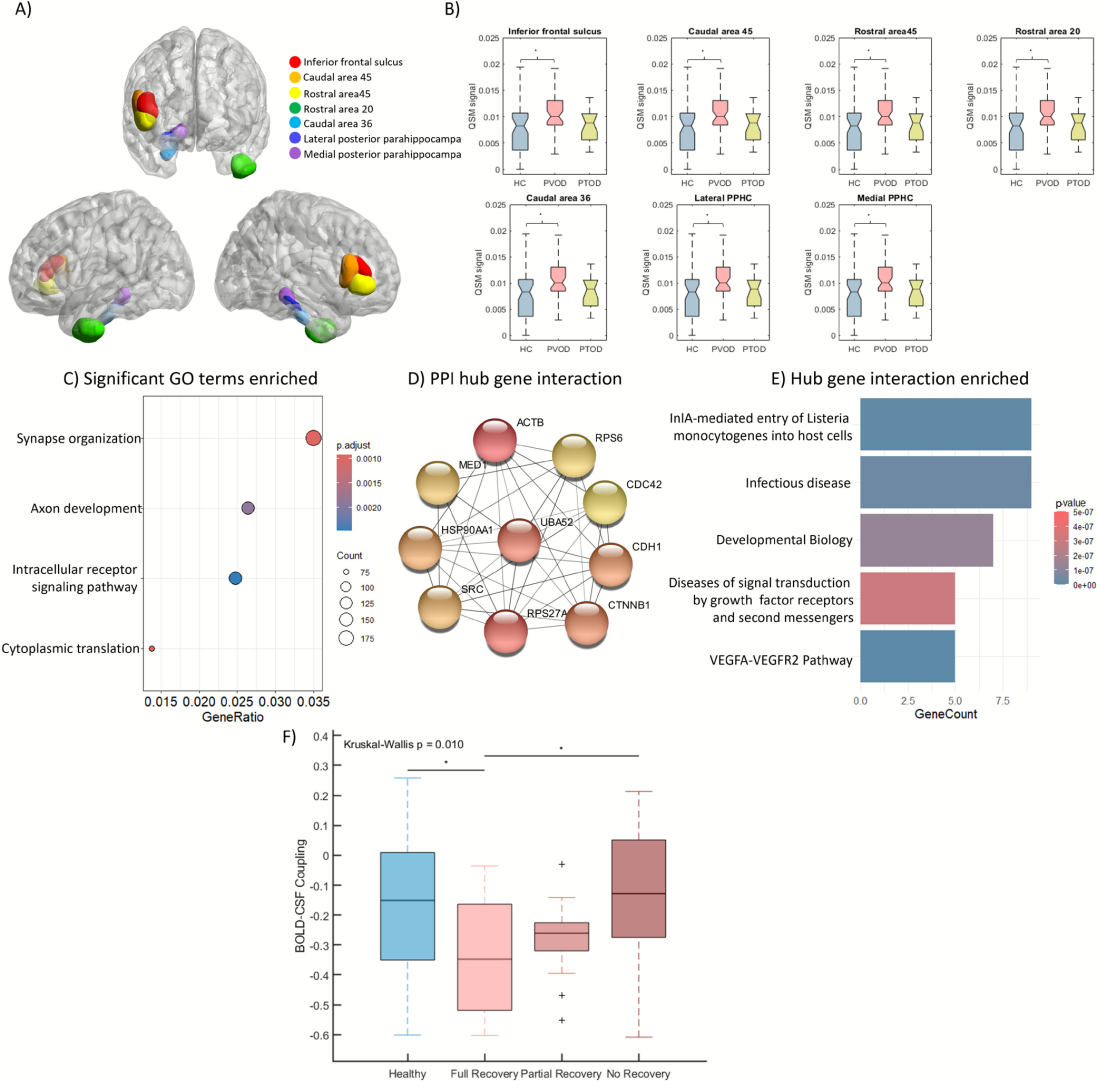
(A) Brain regions with altered QSM values mapped to the BNA‐246 atlas. Significant brain regions are represented by different colors. Red, orange, yellow, green, blue, dark blue, and purple represent the inferior frontal sulcus, caudal area 45, rostral area 45, rostral area 20, caudal area 36, lateral posterior parahippocampal gyrus, and medial posterior parahippocampal gyrus, respectively. (B) One‐way ANOVA comparisons of QSM signal across HC, PTOD, and PVOD among seven significant altered regions. Error bars represent the standard deviation (SD). Blue, red, and yellow represent HC, PVOD, and PTOD. The asterisks represent the significant difference groups. (C) Dot plot of significant GO term enrichment. The color of the dot plot represents the gene enrichment *p*‐value for the specified GO term. The size of the dots represents the number of genes involved in the pathway. (D) Network maps of hub gene interactions among the significant weighted gene sets made by cytoHubba plugin. (E) Bar chat of hub gene interaction enrichment. The color of the dot plot represents the gene enrichment *p*‐value for the specified GO term. The size of the dots represents the number of genes involved in the pathway. (F) Kruskal–Wallis tests compare the BOLD‐CSF coupling of HC, full recovery PVOD, partial recovery PVOD, and no recovery PVOD. Our results show a significant difference in coupling strength between full recovery PVOD and HC (*p* = 0.03) and unrecovered PVOD (*p* = 0.033). Blue, light red, red, and dark red colors represent HC, full recovery PVOD, partial recovery PVOD, and no recovery PVOD. Asterisks represent significant differences between groups.

With sex and age as covariates, regional QSM results demonstrated that PVOD patients had higher brain iron content than the other groups in 6 ROIs, including the inferior frontal gyrus, inferior temporal gyrus, and parahippocampal gyrus. Specifically, PVOD patients had higher brain iron content in the right inferior frontal sulcus, right caudal area (35/36/45), right rostral area (20/45), and right lateral posterior parahippocampal gyrus (FDR‐adjusted, *ps* < 0.05). Figure 3 showed the differences in QSM signal on the BNA246 across three groups.

### 3.4 Transcriptomic Correlation in OD

In the PLS regression result, the highest proportion of QSM changes among gene expressions was 30.13% (*p*_boot_ < 0.01). The selected PLS component was normalized and ranked according to univariate one‐sample *Z* tests. A total of 6276 weighted genes were screened for significant correlation between gene expression and regional variation in QSM (*Z*‐score within ±2.8, *p*_fdr_ < 0.05). The gene with the highest positive weight was the PKIA, which encoded cAMP‐dependent protein kinase inhibitor alpha protein. The gene with the lowest negative weight was the Ubiquitin‐related modifier‐1 (URM1), as a protein tag involved in the urmylation of proteins involved in nutrient sensing and the oxidative stress response.

### 3.5 Gene Set Enrichment and PPI Results

After performing GO enrichment analysis, GO terms—biological pathways—were found and broadly mapped to neuron development, including “axon development,” “synapse organization,” “intracellular receptor signaling pathway,” and “cytoplasmic translation” (Figure 3C). Besides, the neural‐related GO biological process is enriched for PLS component genes, whereas the KEGG pathway was not. Significant sets of genes were further mapped to PPI networks. The network was filtered with a threshold interaction score of 0.7 and the hub genes were selected using cytoHubba. A total of 10 nodes and 38 edges of the hub gene network were extracted (Figure 3D). Functional enrichment analysis revealed 42 significant pathways (*p* < 0.001, FDR corrected). Of these, we focused on the top five important biological pathways, including “Diseases of signal transduction by growth factors and second messengers,” “VEGFA‐VEGFAR2 pathway” among others (Figure 3E).

### 3.6 Alteration in BOLD‐CSF Coupling After PVOD Prognosis

Among 48 patients who completed the telephone follow‐up, 37 of them provided recovery status information, while the rest did not reply, possibly due to personal reluctance or other reasons. The distribution of outcomes was as follows: partial recovery (*n* = 12), complete recovery (*n* = 16), and no recovery (*n* = 9). A subset of 16 patients participated in on‐site visits with TDI testing that were distributed as follows: partial recovery (*n* = 5), complete recovery (*n* = 7), and no recovery (*n* = 4). The recovery distribution and clinical characteristics are summarized in Table S1. To further investigate the BOLD‐CSF changes in the three prognostic stages of PVOD, we first conducted a Spearman correlation study between BOLD‐CSF coupling and prognostic stages, which showed a significant positive correlation between the two (Spearman's *r* = 0.369, *p* = 0.011). Subsequently, the Kruskal–Wallis test was used to compare the BOLD‐CSF coupling of the four groups (HC, full recovery PVOD, partial recovery PVOD and no recovery PVOD) and a significant difference was found (chi‐sq = 11.43, *p* = 0.0096). Post hoc test showed significantly lower coupling strength in full recovery PVOD compared to the HC group (*p* = 0.03) and no recovery PVOD (*p* = 0.033), with no significant differences between other group comparisons (*ps* > 0.05) (Figure 3F).

### 3.7 Clinical Characteristic Associated With BOLD‐CSF Coupling and QSM Signal

We further investigated the association between clinical characteristics and neuroimaging indicators (BOLD‐CSF coupling, QSM) across all subjects (HC, PVOD, PTOD) and three PVOD stages (full recovery, partial recovery and no recovery). However, our results revealed no significant correlation between neuroimaging indicators and SS total score in any subgroup (FDR‐adjusted, *ps* < 0.05, see Figures S3 and S4).

## 4 Discussion

The present study evaluated changes in brain function in patients with OD through glymphatic function and dysregulated iron accumulation using multiparametric MRI and gene expression techniques. By integrating gene expression data, pathways influencing metal ion transport in the brains of OD patients were explored at a microscopic level. Notably, cortical glymphatic movements were significantly stronger in PVOD than in HC, as measured by BOLD‐CSF coupling. Additionally, by comparing the prognosis of PVOD, it was found that the glymphatic movement of fully recovered PVOD was significantly stronger than that of HC and did not recover. The regional increases in magnetic susceptibility also reflected higher iron accumulation in PVOD. Linked gene expressions predominantly mapped to neuronal development pathways, including axon development and synapse organization.

BOLD‐CSF coupling has been shown to be weaker in neurodegenerative diseases such as Alzheimer's disease or Parkinson's disease [17, 43]. In contrast to previous evidence, our results showed the opposite result, with the coupling of BOLD‐CSF flow being stronger in PVOD. In the prognostic categorization, fully restored BOLD‐CSF flows are more strongly coupled than unrestored BOLD‐CSF flows and trends from unrestored to partially restored to fully restored. The coupling between BOLD and CSF signals reflected the equilibrium between hemoglobin metabolism and the clearance neurotoxin process during metabolism in the brain [17]. In neurodegenerative patients, the slowing down of glymphatic movement due to neurodegeneration led to decreased clearance capability and inflammation accumulation in the cerebral cortex. In PVOD, the results of glymphatic function showed the opposite performance, especially in the fully restored PVOD group. We can propose a possible explanation that after viral invasion of the brain, the brain compensates by enhancing the clearance of pathological proteins, improving the oxidative stress environment, increasing neuroplasticity, and reducing neuronal death. This improved clearance mechanism suggests the involvement of clearance mechanisms in the functional regulation of the brain in infectious diseases.

A notable finding of this study was the increased iron accumulation in the right subcortical region of PVOD patients. Abnormal accumulation of iron generates hydroxyl radicals that trigger oxidative stress, which in turn causes tissue damage and neuronal loss [22]. Regions such as the right inferior frontal sulcus, right caudal area (35/36/45), right rostral area (20/45), and the right lateral posterior parahippocampal gyrus were identified as focal points of neurotoxicity and neurodegeneration. These regions are thought to aid in olfactory memory and recognition. They combine sensory input with memory to help recognize familiar odors and associate them with past experiences [44, 45, 46]. While these regions are not primary olfactory functions, they are integral to the higher‐order processing of smells. High QSM signals in these regions are likely to indicate the accumulation of inflammation and tissue changes in PVOD patients, which progressively leads to impaired recognition and memory of smells in the PVOD. Yet, QSM and BOLD‐CSF coupling were not significantly different in the PTOD from the HC group. This may be due to differences in condition or other factors. According to the clinical scales, the olfactory degradation was lower in PTOD than in PVOD, which may explain the lack of significant neuroimaging differences. Another possibility is that the difference in the mechanism of olfactory alteration between PVOD and PTOD is such that the exogenous impairment does not manifest itself in the removal and accumulation of toxins, but rather in the direct expression of structural impairment or alteration.

Using PLS regression to integrate iron accumulation patterns from neural images with genetic data, significant genes associated with high iron deposition in PVOD patients were identified. These genes were highly expressed in regions with elevated iron accumulation in PVOD patients. According to GO analysis, these genes exemplify biological pathways important for neuronal development. Specifically, we found that the weighted genes showed significantly greater expression in axon development and synapse organization. The olfactory function begins with the encoding of odors through axons of olfactory neurons, which express specific receptor proteins in the olfactory bulb. These neurons synapse with mitral or tufted cells, facilitating further signal transmission [47]. Previous studies have suggested that secondary damage to synaptic plasticity in combination with initial viral damage is one of the conditions necessary for the clinical development of PVOD [48]. Neurotoxic viruses are capable of altering synaptic plasticity by unknown mechanisms, and synaptic plasticity in neurons undergoes long‐lasting changes even after viral clearance. A similar picture emerges for other virus‐infected diseases, such as influenza A and RSV infections [49, 50].

Additionally, our results revealed significant enrichment in the “intracellular receptor signaling pathway” and “cytoplasmic translation.” Cytoplasmic translation is the process of synthesizing proteins. Proteins synthesized near synapses are essential for synaptic plasticity and are tightly regulated by intracellular receptor signaling pathways [51]. When intracellular receptor signaling pathways are activated, these receptors initiate intracellular signaling cascades involving molecules such as kinases, phosphatases, and secondary messengers [52]. Furthermore, our PPI results showed functional enrichment of “growth factor and second messenger signaling diseases” and “VEGFA‐VEGFAR2 pathway,” which also provides evidence that secondary signaling cascades are amplified and propagated to regulate cell survival and cellular function. These secondary signaling pathways (e.g., the MAPK/ERK pathway or the mTOR pathway) may drive changes in gene expression and protein synthesis, resulting in lasting changes in synaptic plasticity [53]. These molecular pathways may reflect a coordinated biological response aimed at restoring neuronal structure and function following viral insult.

Collectively, these findings indicate that PVOD triggers a neuroinflammatory environment marked by increased iron accumulation in specific brain regions, leading to cellular dysfunction and neuronal death. In response, the brain activates biological pathways to enhance neuroplasticity and neuronal organization. Simultaneously, enhanced glymphatic function facilitates the clearance of inflammatory substances, ameliorating the neuroinflammatory environment. While this interpretation remains hypothetical, the observed convergence of glymphatic enhancement, iron dysregulation, and plasticity‐related gene expression provides a plausible framework for understanding how the brain might adapt to viral‐induced OD.

### 4.1 Limitations

There are several limitations in the present study. The study did not include a longitudinal analysis of PVOD patients recovering from the disorder, which could provide critical insights into the hypothesized mechanisms of recovery. Although we had follow‐up data on a subset of PVOD patients, these analyses were limited by the dropout and insufficient number of subjects' follow‐up visits. Future studies could add more complete follow‐up and neuroimaging data. Another limitation lies in the use of gene expression profiles derived from healthy individuals. This introduces an important interpretational caveat, as the gene expression profiles may not accurately reflect disease‐specific alterations in PVOD patients. Our analyses were limited to comparisons between HC and PVOD patients, without direct evidence of PVOD‐specific transcriptional changes. Future studies can improve the specificity of PVOD by adding more PVOD‐related bioinformatic analyses to enhance our understanding of OD.

## 5 Conclusions

In summary, this study represents a novel application of multimodal methodologies to investigate glymphatic function and its relationship with iron accumulation and gene expression in olfactory disorders. The findings revealed enhanced glymphatic function in PVOD, alongside evidence of iron accumulation in the right inferior cortical regions. By integrating PLS analysis of gene expression and neuroimaging data, the study identified genes and biological pathways associated with OD, particularly those involved in neuronal organization.

Functional enrichment analysis highlighted pathways linked to iron accumulation, focusing primarily on neuronal organization and synaptic plasticity. These results suggest that neurotoxic effects can alter olfactory memory and recognition processes. Additionally, the observed enhancement of glymphatic function and activation of neuroplasticity‐related biological pathways may contribute to ameliorating OD. Collectively, the findings provide new insights into the neurobiological mechanisms underlying olfactory diseases. These findings also suggest the potential for using neurotoxins as biomarkers for OD.

## Author Contributions

C.L. analyzed and interpreted patient data regarding olfactory dysfunction and made significant contributions to writing the manuscript and figures/tables. Z.Y. and G.M. were responsible for the study project, revision of the article, and grant support. J.L. was responsible for data collection and participated in some of the data analysis. All authors read and approved the final manuscript.

## Funding

This study was supported by the National Natural Science Foundation of China (No. U25A20136, 82271953), the Open Research Fund of the State Key Laboratory of Cognitive Neuroscience and Learning (No. CNLZD2303), the STI2030‐Major Projects (No. 2022ZD0213300), the University of Macau (MYRG2022‐00054‐FHS, MYRG‐GRG2023‐00038‐FHS‐UMDF, MYRG‐GRG2024‐00259‐FHS), the Macao Science and Technology Development Fund (FDCT 0014/2024/RIB1).

## Ethics Statement

This study was approved by the Ethics Committee of the China‐Japan Friendship Hospital's institute (No. 2022‐KY‐181) and conducted in accordance with the Declaration of Helsinki. The participants were informed and they agreed to participate in this study.

## Conflicts of Interest

The authors declare no conflicts of interest.

## Supporting information


**Data S1:** cns70677‐sup‐0001‐DataS1.xlsx.

**Appendix S1:** cns70677‐sup‐0002‐AppendixS1.docx.

## Data Availability

The raw data used in the preparation of this article were obtained from the China‐Japan Friendship Hospital. Where ethical criteria are satisfied, data are available on request from the correspondence authors. Processed data are accessible in the Data S1. Open‐source software was used for all analyses. Code used in the analyses described in this paper is available before publication.

## References

[1] R. L. Doty , “Clinical Disorders of Olfaction,” in Handbook of Olfaction and Gustation (Wiley, 2015), 375–402.

[2] A. Pokharel , “Olfactory Dysfunction: A Clinical Marker of COVID‐19,” JNMA; Journal of the Nepal Medical Association 59, no. 233 (2021): 88–93. 34508447 10.31729/jnma.5658 PMC7893401

[3] J. D. Mainland , E. A. Bremner , N. Young , et al., “One Nostril Knows What the Other Learns,” Nature 419, no. 6909 (2002): 802. 10.1038/419802a 12397347

[4] D. Wilson , A. Best , and R. Sullivan , “Plasticity in the Olfactory System: Lessons for the Neurobiology of Memory,” Neuroscientist 10, no. 6 (2004): 513–524. 15534037 10.1177/1073858404267048 PMC1868530

[5] I. Konstantinidis , E. Tsakiropoulou , P. Bekiaridou , C. Kazantzidou , and J. Constantinidis , “Use of Olfactory Training in Post‐Traumatic and Postinfectious Olfactory Dysfunction,” Laryngoscope 123, no. 12 (2013): E85–E90. 24114690 10.1002/lary.24390

[6] M. Damm , L. K. Pikart , H. Reimann , et al., “Olfactory Training Is Helpful in Postinfectious Olfactory Loss: A Randomized, Controlled, Multicenter Study,” Laryngoscope 124, no. 4 (2014): 826–831. 23929687 10.1002/lary.24340

[7] R. Pellegrino , A. Hahner , V. Bojanowski , C. Hummel , J. Gerber , and T. Hummel , “Olfactory Function in Patients With Hyposmia Compared to Healthy Subjects‐An fMRI Study,” Rhinology 54, no. 4 (2016): 374–381. 27421303 10.4193/Rhin16.098

[8] P. Han , N. Winkler , C. Hummel , A. Hähner , J. Gerber , and T. Hummel , “Impaired Brain Response to Odors in Patients With Varied Severity of Olfactory Loss After Traumatic Brain Injury,” Journal of Neurology 265 (2018): 2322–2332. 30109478 10.1007/s00415-018-9003-8

[9] M. S. Xydakis , M. W. Albers , E. H. Holbrook , et al., “Post‐Viral Effects of COVID‐19 in the Olfactory System and Their Implications,” Lancet Neurology 20, no. 9 (2021): 753–761. 34339626 10.1016/S1474-4422(21)00182-4 PMC8324113

[10] L. Muccioli , G. Sighinolfi , M. Mitolo , et al., “Cognitive and Functional Connectivity Impairment in Post‐COVID‐19 Olfactory Dysfunction,” NeuroImage: Clinical 38 (2023): 103410. 37104928 10.1016/j.nicl.2023.103410 PMC10165139

[11] L. Bonanno , S. Marino , S. de Salvo , et al., “Role of Diffusion Tensor Imaging in the Diagnosis and Management of Post‐Traumatic Anosmia,” Brain Injury 31, no. 13–14 (2017): 1964–1968. 28816545 10.1080/02699052.2017.1346293

[12] P. Nigro , A. Chiappiniello , S. Simoni , et al., “Changes of Olfactory Tract in Parkinson's Disease: A DTI Tractography Study,” Neuroradiology 63 (2021): 235–242. 32918150 10.1007/s00234-020-02551-4

[13] D. Güllmar , T. Seeliger , H. Gudziol , et al., “Improvement of Olfactory Function After Sinus Surgery Correlates With White Matter Properties Measured by Diffusion Tensor Imaging,” Neuroscience 360 (2017): 190–196. 28797663 10.1016/j.neuroscience.2017.07.070

[14] M. Leon , E. T. Troscianko , and C. C. Woo , “Inflammation and Olfactory Loss Are Associated With at Least 139 Medical Conditions,” Frontiers in Molecular Neuroscience 17 (2024): 1455418. 39464255 10.3389/fnmol.2024.1455418 PMC11502474

[15] Y. Cai , Y. Zhang , S. Leng , et al., “The Relationship Between Inflammation, Impaired Glymphatic System, and Neurodegenerative Disorders: A Vicious Cycle,” Neurobiology of Disease 192 (2024): 106426. 38331353 10.1016/j.nbd.2024.106426

[16] N. E. Fultz , G. Bonmassar , K. Setsompop , et al., “Coupled Electrophysiological, Hemodynamic, and Cerebrospinal Fluid Oscillations in Human Sleep,” Science 366, no. 6465 (2019): 628–631. 31672896 10.1126/science.aax5440 PMC7309589

[17] F. Han , J. Chen , A. Belkin‐Rosen , et al., “Reduced Coupling Between Cerebrospinal Fluid Flow and Global Brain Activity Is Linked to Alzheimer Disease–Related Pathology,” PLoS Biology 19, no. 6 (2021): e3001233. 34061820 10.1371/journal.pbio.3001233 PMC8168893

[18] F. Han , J. Q. Lee , X. Chen , et al., “Reduced Coupling Between Cerebrospinal Fluid Flow and Global Brain Activity Is Linked to Tau Pathology,” Alzheimer's & Dementia 19 (2023): e075860.

[19] Z. Wang , Z. Song , C. Zhou , et al., “Reduced Coupling of Global Brain Function and Cerebrospinal Fluid Dynamics in Parkinson's Disease,” Journal of Cerebral Blood Flow & Metabolism 43, no. 8 (2023): 1328–1339. 36927139 10.1177/0271678X231164337 PMC10369155

[20] L. Wu , Z. Zhang , X. Liang , et al., “Glymphatic System Dysfunction in Recovered Patients With Mild COVID‐19: A DTI‐ALPS Study,” iScience 27, no. 1 (2024): 108647. 38155770 10.1016/j.isci.2023.108647 PMC10753064

[21] L. Deng , Q. Luo , Y. Liu , et al., “Progressive Iron Overload in Middle‐Aged Mice Impairs Olfactory Function, Triggers Lipid Oxidation and Induces Apoptosis,” Frontiers in Pharmacology 15 (2024): 1506944. 39749201 10.3389/fphar.2024.1506944 PMC11693683

[22] M. Conrad , J. P. F. Angeli , P. Vandenabeele , and B. R. Stockwell , “Regulated Necrosis: Disease Relevance and Therapeutic Opportunities,” Nature Reviews Drug Discovery 15, no. 5 (2016): 348–366. 26775689 10.1038/nrd.2015.6 PMC6531857

[23] S. J. Dixon , K. M. Lemberg , M. R. Lamprecht , et al., “Ferroptosis: An Iron‐Dependent Form of Nonapoptotic Cell Death,” Cell 149, no. 5 (2012): 1060–1072. 22632970 10.1016/j.cell.2012.03.042 PMC3367386

[24] T. Grubić Kezele and B. Ćurko‐Cofek , “Age‐Related Changes and Sex‐Related Differences in Brain Iron Metabolism,” Nutrients 12, no. 9 (2020): 2601. 32867052 10.3390/nu12092601 PMC7551829

[25] C. Liu , H. Wei , N. J. Gong , M. Cronin , R. Dibb , and K. Decker , “Quantitative Susceptibility Mapping: Contrast Mechanisms and Clinical Applications,” Tomography 1, no. 1 (2015): 3–17. 26844301 10.18383/j.tom.2015.00136 PMC4734903

[26] A. Arnatkeviciute , B. D. Fulcher , M. A. Bellgrove , and A. Fornito , “Imaging Transcriptomics of Brain Disorders,” Biological Psychiatry Global Open Science 2, no. 4 (2022): 319–331. 36324650 10.1016/j.bpsgos.2021.10.002 PMC9616271

[27] J. Seidlitz , A. Nadig , S. Liu , et al., “Transcriptomic and Cellular Decoding of Regional Brain Vulnerability to Neurogenetic Disorders,” Nature Communications 11, no. 1 (2020): 3358. 10.1038/s41467-020-17051-5 PMC7335069 32620757

[28] J. Zheng , F. Y. Womer , L. Tang , et al., “Integrative Omics Analysis Reveals Epigenomic and Transcriptomic Signatures Underlying Brain Structural Deficits in Major Depressive Disorder,” Translational Psychiatry 14, no. 1 (2024): 17. 38195555 10.1038/s41398-023-02724-8 PMC10776753

[29] A. Fornito , A. Arnatkevičiūtė , and B. D. Fulcher , “Bridging the Gap Between Connectome and Transcriptome,” Trends in Cognitive Sciences 23, no. 1 (2019): 34–50. 30455082 10.1016/j.tics.2018.10.005

[30] C. Zhang , H. Cai , X. Xu , et al., “Genetic Architecture Underlying Differential Resting‐State Functional Connectivity of Subregions Within the Human Visual Cortex,” Cerebral Cortex 32, no. 10 (2022): 2063–2078. 34607357 10.1093/cercor/bhab335

[31] C.‐G. Yan , X. D. Wang , X. N. Zuo , and Y. F. Zang , “DPABI: Data Processing & Analysis for (Resting‐State) Brain Imaging,” Neuroinformatics 14 (2016): 339–351. 27075850 10.1007/s12021-016-9299-4

[32] J. Ashburner , G. Barnes , C. C. Chen , et al., SPM12 Manual, vol. 2464, no. 4 (Wellcome Trust Centre for Neuroimaging, 2014).

[33] K. M. Aquino , B. D. Fulcher , L. Parkes , K. Sabaroedin , and A. Fornito , “Identifying and Removing Widespread Signal Deflections From fMRI Data: Rethinking the Global Signal Regression Problem,” NeuroImage 212 (2020): 116614. 32084564 10.1016/j.neuroimage.2020.116614

[34] L. Fan , H. Li , J. Zhuo , et al., “The Human Brainnetome Atlas: A New Brain Atlas Based on Connectional Architecture,” Cerebral Cortex 26, no. 8 (2016): 3508–3526. 27230218 10.1093/cercor/bhw157 PMC4961028

[35] W. Li , A. V. Avram , B. Wu , X. Xiao , and C. Liu , “Integrated Laplacian‐Based Phase Unwrapping and Background Phase Removal for Quantitative Susceptibility Mapping,” NMR in Biomedicine 27, no. 2 (2014): 219–227. 24357120 10.1002/nbm.3056 PMC3947438

[36] B. Wu , W. Li , A. Guidon , and C. Liu , “Whole Brain Susceptibility Mapping Using Compressed Sensing,” Magnetic Resonance in Medicine 67, no. 1 (2012): 137–147. 21671269 10.1002/mrm.23000 PMC3249423

[37] H. Wei , R. Dibb , Y. Zhou , et al., “Streaking Artifact Reduction for Quantitative Susceptibility Mapping of Sources With Large Dynamic Range,” NMR in Biomedicine 28, no. 10 (2015): 1294–1303. 26313885 10.1002/nbm.3383 PMC4572914

[38] M. J. Hawrylycz , E. S. Lein , A. L. Guillozet‐Bongaarts , et al., “An Anatomically Comprehensive Atlas of the Adult Human Brain Transcriptome,” Nature 489, no. 7416 (2012): 391–399. 22996553 10.1038/nature11405 PMC4243026

[39] A. Arnatkevic̆iūtė , B. D. Fulcher , and A. Fornito , “A Practical Guide to Linking Brain‐Wide Gene Expression and Neuroimaging Data,” NeuroImage 189 (2019): 353–367. 30648605 10.1016/j.neuroimage.2019.01.011

[40] S. E. Morgan , J. Seidlitz , K. J. Whitaker , et al., “Cortical Patterning of Abnormal Morphometric Similarity in Psychosis Is Associated With Brain Expression of Schizophrenia‐Related Genes,” Proceedings of the National Academy of Sciences of the United States of America 116, no. 19 (2019): 9604–9609. 31004051 10.1073/pnas.1820754116 PMC6511038

[41] K. J. Whitaker , P. E. Vértes , R. Romero‐Garcia , et al., “Adolescence Is Associated With Genomically Patterned Consolidation of the Hubs of the Human Brain Connectome,” Proceedings of the National Academy of Sciences of the United States of America 113, no. 32 (2016): 9105–9110. 27457931 10.1073/pnas.1601745113 PMC4987797

[42] M. E. Smoot , K. Ono , J. Ruscheinski , P. L. Wang , and T. Ideker , “Cytoscape 2.8: New Features for Data Integration and Network Visualization,” Bioinformatics 27, no. 3 (2011): 431–432. 21149340 10.1093/bioinformatics/btq675 PMC3031041

[43] F. Han , G. L. Brown , Y. Zhu , et al., “Decoupling of Global Brain Activity and Cerebrospinal Fluid Flow in Parkinson's Disease Cognitive Decline,” Movement Disorders 36, no. 9 (2021): 2066–2076. 33998068 10.1002/mds.28643 PMC8453044

[44] J. A. Gottfried , A. P. R. Smith , M. D. Rugg , and R. J. Dolan , “Remembrance of Odors Past: Human Olfactory Cortex in Cross‐Modal Recognition Memory,” Neuron 42, no. 4 (2004): 687–695. 15157428 10.1016/s0896-6273(04)00270-3

[45] D. A. Wilson and R. M. Sullivan , “Cortical Processing of Odor Objects,” Neuron 72, no. 4 (2011): 506–519. 22099455 10.1016/j.neuron.2011.10.027 PMC3223720

[46] A. Arshamian , E. Iannilli , J. C. Gerber , et al., “The Functional Neuroanatomy of Odor Evoked Autobiographical Memories Cued by Odors and Words,” Neuropsychologia 51, no. 1 (2013): 123–131. 23147501 10.1016/j.neuropsychologia.2012.10.023

[47] R. Choi and B. J. Goldstein , “Olfactory Epithelium: Cells, Clinical Disorders, and Insights From an Adult Stem Cell Niche,” Laryngoscope Investigative Otolaryngology 3, no. 1 (2018): 35–42. 29492466 10.1002/lio2.135 PMC5824112

[48] J. C. Lee , R. Nallani , L. Cass , V. Bhalla , A. G. Chiu , and J. A. Villwock , “A Systematic Review of the Neuropathologic Findings of Post‐Viral Olfactory Dysfunction: Implications and Novel Insight for the COVID‐19 Pandemic,” American Journal of Rhinology & Allergy 35, no. 3 (2021): 323–333. 32915650 10.1177/1945892420957853 PMC10404900

[49] S. Hosseini , E. Wilk , K. Michaelsen‐Preusse , et al., “Long‐Term Neuroinflammation Induced by Influenza A Virus Infection and the Impact on Hippocampal Neuron Morphology and Function,” Journal of Neuroscience 38, no. 12 (2018): 3060–3080. 29487124 10.1523/JNEUROSCI.1740-17.2018 PMC6596076

[50] J. A. Espinoza , K. Bohmwald , P. F. Céspedes , et al., “Impaired Learning Resulting From Respiratory Syncytial Virus Infection,” Proceedings of the National Academy of Sciences of the United States of America 110, no. 22 (2013): 9112–9117. 23650398 10.1073/pnas.1217508110 PMC3670318

[51] C. Wu and D. Sun , “GABA Receptors in Brain Development, Function, and Injury,” Metabolic Brain Disease 30 (2015): 367–379. 24820774 10.1007/s11011-014-9560-1 PMC4231020

[52] M. A. Robichaux and C. W. Cowan , “Signaling Mechanisms of Axon Guidance and Early Synaptogenesis,” in The Neurobiology of Childhood (Springer, 2014), 19–48. 10.1007/7854_2013_255 24318963

[53] I. Farhy‐Tselnicker and N. J. Allen , “Astrocytes, neurons, synapses: a tripartite view on cortical circuit development,” Neural Development 13 (2018): 1–12. 29712572 10.1186/s13064-018-0104-y PMC5928581

