# Supplementary material for: Neuroimaging and Transcriptomic Insights Into Iron Accumulation and Glymphatic Dysfunction in Olfactory Dysfunction
Source: CNS Neurosci Ther. 2026 Jan 16;32(1):e70677. doi: 10.1002/cns.70677 (PMC12811075; doi:10.1002/cns.70677)
Supplement: Supplementary file 2 — Appendix S1: cns70677‐sup‐0002‐AppendixS1.docx. [file CNS-32-e70677-s002.docx]

**Supporting Information**

[Quality Control 1](#_Toc206072026)

[Figure S1. 1](#_Toc206072027)

[Confounder effects in BOLD-CSF coupling alteration 2](#_Toc206072028)

[Figure S2. 2](#_Toc206072029)

[Clinical characteristic correlated with BOLD-CSF coupling and QSM signal 2](#_Toc206072030)

[Figure S3. 3](#_Toc206072031)

[Supporting Information Table 1. Clinical Outcomes of PVOD Patients 3](#_Toc206072032)

[Clinical characteristics in relation to BOLD-CSF coupling of PVOD in prognostic recovery stages 4](#_Toc206072033)

[Figure S4. 5](#_Toc206072034)

[References 5](#_Toc206072035)

**Quality Control**

To ensure the quality of the imaging data, rs-fMRI and QSM data that showed excessive motion, artifactual images, or poor brain coverage were excluded. For rs-fMRI data, an outlier test was performed for each subject, in which frame displacement (FD) was estimated using the Friston 24-parameter motion correction implemented in the DPABI software. FD was the cumulative sum of the absolute values of all six translational and rotational correction parameters (Yan et al., 2016). Any volume with FD > 0.2 mm, as well as 2 volumes in front and 1 volume behind these masses, can be labeled as a bad time point. Subjects with an average volume FD > 0.2 mm or more that are labeled as rejected frames will be excluded from further analysis. Subjects ultimately included in the analysis included 41 in the HC (9 excluded) and 48 in the PVOD (8 excluded). In addition to these automated quality control measures, all pre-processed rs-fMRI time series were visually inspected for artifacts and CSFs that could affect the bottom slice of the rs-fMRI data. For QSM, the pre-processed QSM images were manually inspected and excluded if they were deformed or distorted. Furthermore, to explore the head motion effect in BOLD-CSF coupling, the BOLD-CSF coupling strength was correlated with the FD value. The BOLD-CSF coupling strength did not show a significant correlation with the head motion of subjects (*r* = -0.123, *p* = 0.206; Figure S1)


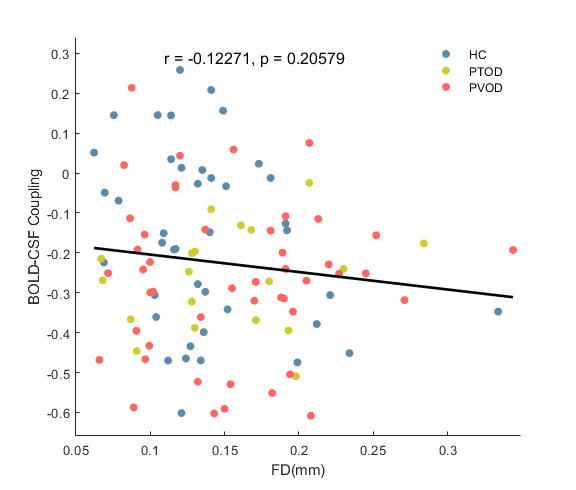


**Figure S1.**

**The head motion did not affect the BOLD-CSF coupling strength.** The head motion of subjects, quantified by mean FD, was not correlated with the BOLD-CSF coupling strength (*r* = -0.123, *p* = 0.206).

**Confounder effects in BOLD-CSF coupling alteration**

To investigate the confounder effect in BOLD-CSF coupling alteration, the BOLD-CSF coupling strength was correlated with the duration of OD. The BOLD-CSF coupling strength did not show a significant correlation with subjects’ duration (*r* = -0.093, *p* = 0.454; Figure S2A). Furthermore, we specifically analyzed treatment effects in our PVOD and PTOD, comparing patients who received corticosteroid therapy (n=42) or not (n=26), as well as those who underwent olfactory training (n=51) or not (n=17). Neither comparison revealed significant within-group differences in BOLD-CSF coupling strength (corticosteroid therapy, *p* = 0.965; olfactory training, *p* = 0.808; Figure S2B). These results suggest that the observed BOLD-CSF coupling alterations in PVOD patients are independent of disease duration or these specific therapeutic interventions.


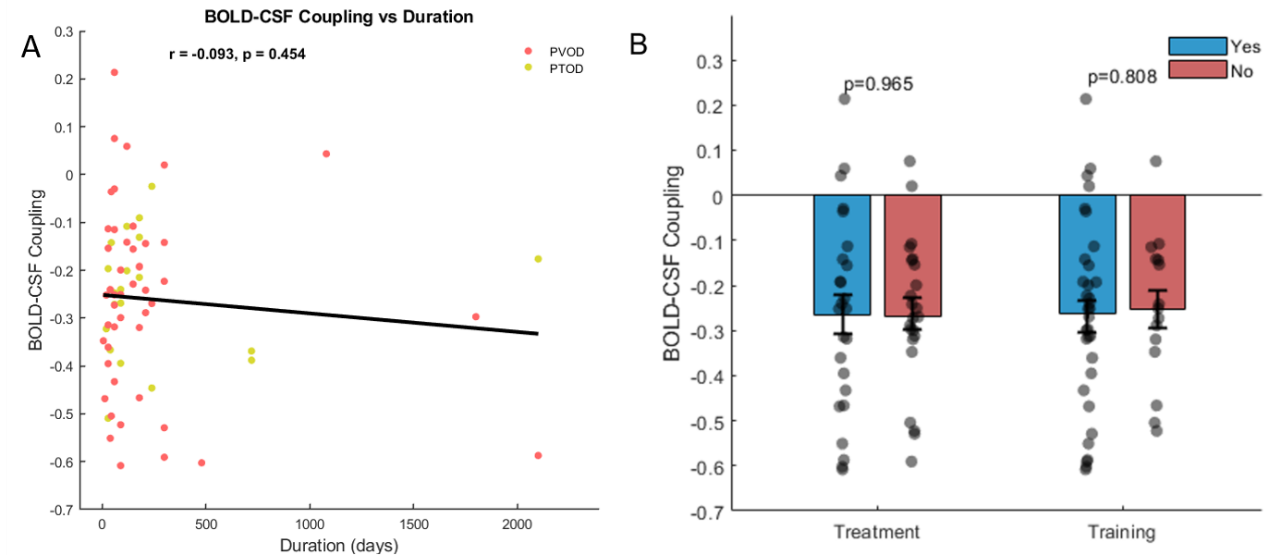


**Figure S2.**

**The strength of BOLD-CSF coupling associated with confounder factors across groups.** (A) Analysis of the temporal coupling strength (quantified by cross-correlation at +3s lag) revealed no significant correlation with disease duration in the overall olfactory dysfunction cohort (*r* = -0.093, *p* = 0.454), with PTOD (yellow) and PVOD (red) patients. (B) Between-group comparisons in coupling strength between healthy controls and OD patients for both corticosteroid therapy and olfactory training interventions. Neither therapeutic approach showed significant effects on coupling strength (Therapy, *p* = 0.965; Training, *p* = 0.808), with comparable values between treated (blue) and untreated (red) patients. Error bars represent standard deviation.

**Clinical characteristic correlated with BOLD-CSF coupling and QSM signal**

We further investigated the correlation between Sniffin' Sticks total score (SS total) and neuroimaging metrics (BOLD-CSF coupling and QSM signals). In both BOLD-CSF coupling and QSM signals, our results showed that there was no significant correlation with SS total score with FDR correction (Fig. S3).


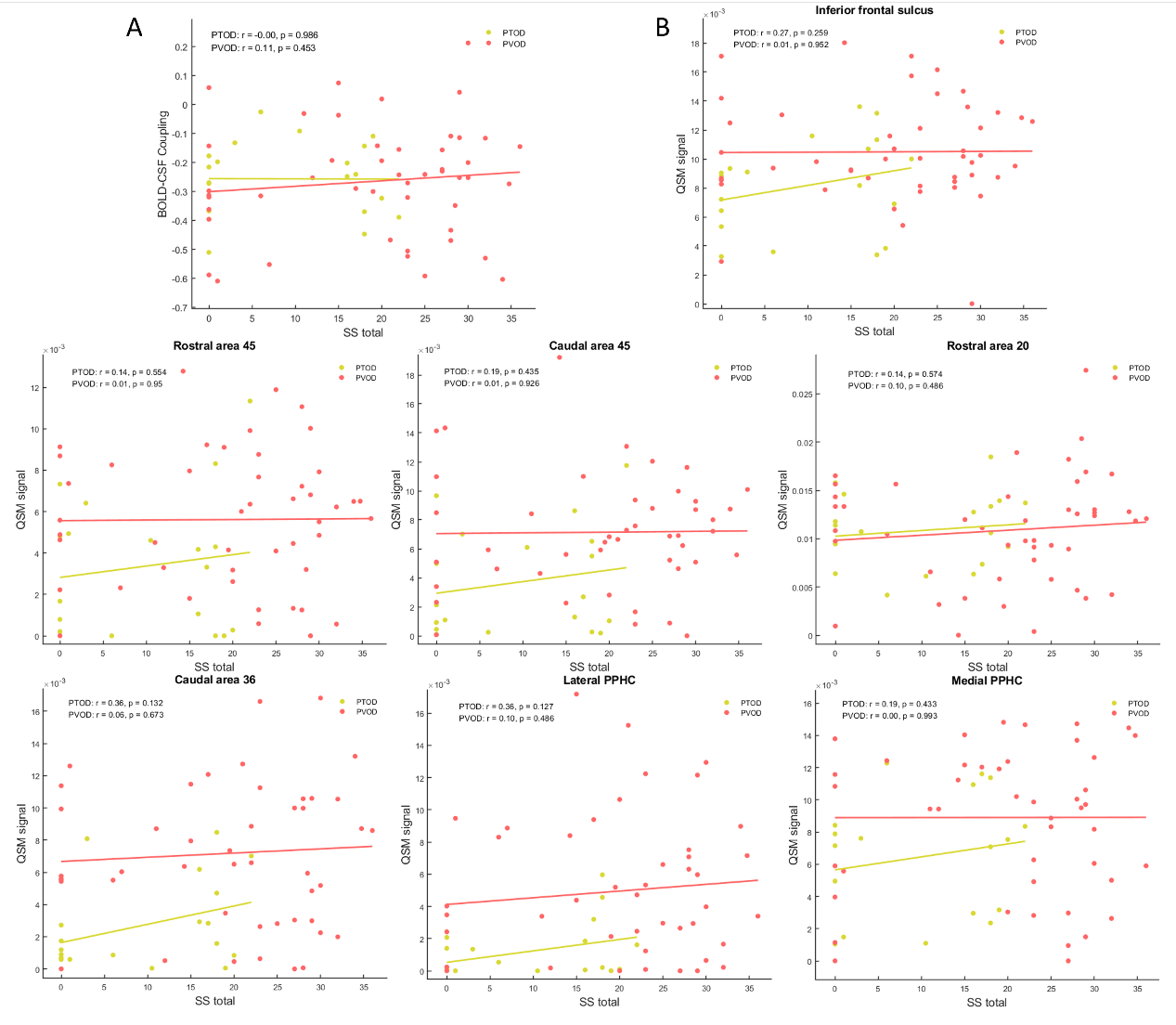


**Figure S3.**

**Correlation of BOLD-CSF coupling and QSM signal strength with different groups of clinical characteristics.** (A) Analysis of BOLD-CSF coupling revealed no significant correlation with SS_total (*r* = 0.11, *p* = 0.453, PVOD; *r* = 0, *p* = 0.986, PTOD). (B) Analysis of QSM signal revealed no significant correlation with SS_total. Specifically, in inferior frontal sulcus (*r* = 0.01, *p* = 0.962, PVOD; *r* = 0.27, *p* = 0.259, PTOD). In rostral area 45 (*r* = 0.01, *p* = 0.95, PVOD; *r* = 0.14, *p* = 0.554, PTOD). In rostral caudal area 45 (*r* = 0.01, *p* = 0.926, PVOD; *r* = 0.19, *p* = 0.435, PTOD). In rostral area 20 (*r* = 0.1, *p* = 0.486, PVOD; *r* = 0.14, *p* = 0.572, PTOD). In caudal area 36 (*r* = 0.06, *p* = 0.673; *r* = 0.36, *p* = 0.132, PTOD). In lateral PPHC (*r* = 0.1, *p* = 0486, PVOD; *r* = 0.36, *p* = 0.127, PTOD). In medial PPHC (*r* = 0, *p* = 0.993, PVOD; *r* = 0.19, *p* = 0.433, PTOD).

**Table 1. Clinical Outcomes of PVOD Patients**

| **Variable** | **Types** | **Baseline** | **Follow-up** |
| --- | --- | --- | --- |
| Etiology | COVID | 34 (49.3%) | / |
|  | PVOD | 35 (50.7%) | / |
| Disease Severity | Anosmia | 23 (33.3%) | 15 (21.7%) |
|  | Hyposmia | 39 (56.5%) | 30 (43.5%) |
|  | Parosmia | 7 (10.2%) | 3 (4.3%) |
|  | VAS Score | 3.25 ± 2.55 | 3.56 ± 2.47 |
| Sniffin' Sticks Test | Threshold (T) | 2.9 ± 2.57 | 3.56 ± 2.65 |
|  | Discrimination (D) | 7.23±4.77 | 6.88 ± 4.09 |
|  | Identification (I) | 8.85±4.99 | 7.56 ± 4.42 |
|  | TDI Total | 18.98±11.2 | 18.06 ± 10.37 |
| Recovery Status | Full recovery | / | 16 (33.3%) |
|  | Partial recovery | / | 12 (25%) |
|  | No recovery | / | 9 (18.8%) |
|  | No response | / | 11 (22.9%) |
| Interventions | Corticosteroid therapy | 25 (75.0%) | / |
|  | Olfactory training | 33 (68.8%) | / |

**Clinical characteristics in relation to BOLD-CSF coupling of PVOD in prognostic recovery stages**

Also, we explored the correlation between SS total and BOLD-CSF coupling across three stages (fully recovery, partial recovery, no recovery) by using Spearman correlation. The results showed that there was no significant correlation between BOLD-CSF coupling and SS total score across three stages (Fig. S4).


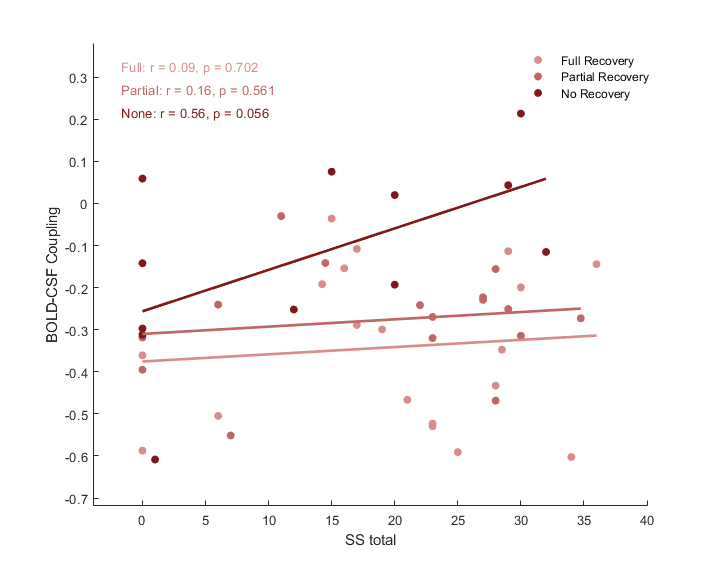


**Figure S4.**

**Correlation of BOLD-CSF coupling with different stages of clinical characteristics in PVOD.** Analysis of BOLD-CSF coupling revealed no significant correlation with SS_total across three stages. In full recovery (*r* = 0.09, *p* = 0.702). In partial recovery (*r* = 0.16, *p* = 0.561). In no recovery (*r* = 0.56, *p* = 0.056).

**References**

Yan, C.-G., Wang, X.-D., Zuo, X.-N., & Zang, Y.-F. (2016). DPABI: data processing & analysis for (resting-state) brain imaging. *Neuroinformatics*, *14*(3), 339-351.
